# The added value of metagenomic next-generation sequencing in central nervous system infections: a systematic review of case reports

**DOI:** 10.1007/s15010-025-02502-2

**Published:** 2025-03-13

**Authors:** Kira Waagner Birkeland, Laurence Mostert, Eric C.J. Claas, Hege Vangstein Aamot, Thomas Demuyser

**Affiliations:** 1 Faculty of Medicine, University of Oslo, Oslo, Norway; 2 Department of Microbiology, Innlandet Hospital Trust, PO Box 104, Brumunddal, Norway; 3 Department of Pathology, Vrije Universiteit Brussel (VUB), Universitair Ziekenhuis Brussel (UZ Brussel), Brussels, Belgium; 4 LUCID-Medical Microbiology and Infection Prevention, Leiden University Medical Center, Leiden, The Netherlands; 5 Department of Microbiology and Infection Control, Akershus University Hospital, Lørenskog, Norway; 6 Department of Nursing, Health and Laboratory Science, Østfold University College, Fredrikstad, Norway; 7 Department of Microbiology, Antwerp University Hospital, Wilrijkstraat 10, Edegem, 2650 Belgium; 8 Department of Bioscience Engineering, University of Antwerp, Groenenborgerlaan 171, Antwerp, 2020 Belgium; 9 AIMS Lab, Center for Neurosciences, Faculty of Medicine and Pharmacy, Vrije Universiteit Brussel (VUB), Brussels, Belgium; 10 Members of ESCMID Study Group for Genomic and Molecular Diagnostics (ESGMD), Brussels, Belgium

**Keywords:** Central nervous system infections, Metagenomic next-generation sequencing, Pathogen indentification, Diagnosis

## Abstract

**Background:**

The diversity of pathogens causing central nervous system (CNS) infections presents a diagnostic challenge. Patient demographics and geographical location affect the likelihood of certain pathogens causing infection. Current diagnostic methods rely on labour-intensive cultivation or targeted detection. Metagenomic next-generation sequencing (mNGS) is a promising tool for detecting pathogens in CNS infections, offering an unbiased approach. To enhance our understanding of patient demographics and the range of pathogens identified through mNGS, we conducted a systematic review of case reports.

**Methods:**

The PubMed database was searched in March 2024. Case reports on CNS infections and mNGS published from January 2014 through February 2024 were included based on predefined criteria.

**Results:**

The search yielded 649 articles, of which 76 were included, encompassing 104 patients. Most patients were male (75%), the median age was 31,5 years [0–75] and 28% were immunocompromised. The most common diagnosis was encephalitis (36%), followed by meningitis (23%) and meningoencephalitis (22%). 53 unique pathogens were identified, comprising 27 different viruses, 19 bacteria, 5 parasites, and 2 fungi. Syndromic encephalitis/meningitis panels would only have detected four of the viruses and five of the bacteria. Additionally, 14 of the bacterial species are considered slow-growing or fastidious and could be challenging to detect by culture.

**Conclusion:**

The application of mNGS in diagnosing CNS infections reveals the diversity of pathogens responsible for these severe infections, thereby improving diagnostics and facilitating targeted treatment. While case reports may be subjected to bias, they provide valuable insights into the use of mNGS in this clinical context.

**Supplementary Information:**

The online version contains supplementary material available at 10.1007/s15010-025-02502-2.

## Background

Infections of the central nervous system (CNS) are a significant cause of morbidity and mortality worldwide. The causative pathogens include bacteria, viruses, fungi and parasites. They can result in a variety of clinical presentations ranging from meningitis and encephalitis to brain abscesses and pathogen-specific infections such as neurosyphilis, cerebral nocardiosis, and neurocysticercosis. Diagnosing CNS infections can be challenging, often requiring a comprehensive approach involving clinical evaluation, elaborate laboratory testing, and brain imaging. However, traditional diagnostic methods may fail to identify the causative agent, resulting in delayed diagnosis or inadequate treatment.

There is a wide variety of pathogens responsible for CNS infections. Factors such as patient characteristics and geographic location can affect what pathogens are most likely to infect the patient. CNS infections can affect people of all ages, but the aetiology can vary among age groups [1]. Immunocompromised and post-neurosurgery patients are particularly vulnerable to CNS infections, and the pathogens involved may differ from those seen in otherwise healthy individuals [2]. Furthermore, the list of possible pathogens expands when considering travel history, seasonal variation and differences in geographic location [3, 4], making it harder for clinicians to diagnose.

Different pathogens exhibit distinct characteristics that can affect the sensitivity and specificity of laboratory tests. While most microbiology labs employ traditional methods such as culture, serology, pathogen-specific PCR and syndromic PCR-panels, culturing fastidious and slow-growing bacteria can be challenging, and targeted methods may fail to detect rare or unexpected pathogens. Moreover, administering antibiotics before collecting specimens can influence the detection rate by culture, making it difficult to provide timely and appropriate treatment to the patient [5].

In recent years, metagenomics next-generation sequencing (mNGS) has emerged as a promising diagnostic tool for identifying the causative pathogen in CNS infections. It allows identifying any pathogen from a single sample [6–9] by using next-generation sequencing of all DNA or RNA in a sample, followed by bioinformatic analysis to identify the microorganism present. This approach can detect common and novel or unexpected pathogens, making it a more comprehensive tool for diagnosing infections.

## Objective

The primary objective of this systematic review is to evaluate the utility of mNGS in diagnosing CNS infections based on existing case reports. We intend to consolidate and analyse these reports to understand better patient demographics and the range of pathogens identified through mNGS, emphasising instances where the technique offered additional diagnostic insights unavailable through traditional laboratory methods. The review aims to contribute to formulating clinical guidelines in this area. Our focus on case reports allows for an in-depth exploration of individual clinical scenarios, offering a more accurate depiction of how mNGS is employed in real-world settings to diagnose complex or atypical infections that may be overlooked in broader studies.

## Methods

This systematic review was conducted according to the PRISMA guidelines [10]. The PubMed database was searched for studies on CNS infections diagnosed with mNGS on the 6th of March 2024. The complete search string can be found in Supplementary Table S1.

The following inclusion and exclusion criteria were applied: case reports of both community-acquired and healthcare-associated infections published between January 2014 through February 2024 were included. The search was not restricted by language. The clinical samples included CSF, brain abscesses and brain biopsies, where nucleic acids were extracted directly from the clinical sample, e.g., not performed on cultured bacteria or viruses. In this review, mNGS is defined as an untargeted sequencing approach that does not enrich for specific species, unlike methods such as hybrid capture, amplicon sequencing, or 16 S/18S/ITS sequencing. Case reports were excluded when mNGS analyses gave no additional information compared to conventional methods performed. Conventional methods entailed cultivation, microscopy, serology and/or targeted molecular analysis. Additionally, articles were excluded if the papers did not describe the methods sufficiently, i.e. it was not possible to determine if untargeted metagenomics sequencing had been performed.

### Data collection

Two reviewers, LM and KWB, independently screened the articles based on title and abstract. Discrepancies were discussed among LM, KWB, ECJC, HVA and TD. Articles eligible for full-text reading were then independently assessed by both LM and KWB. Discrepancies were discussed among LM, KWB, ECJC, HVA and TD.

LM and KWB independently extracted data from the included articles and any discrepancies were checked and corrected. Data from full-text articles included first author, publication year, country of origin, patient gender and age, diagnosis, sample type, pathogen, immune status, pleocytosis, biochemical analyses, travel history and mNGS technology (Supplementary Table S2).

The age of the patients was stratified into five age groups: <1 years, 1–12 years, 13–18 years, 19–65 years and > 65 years. The reported diagnosis was stratified into broader diagnosis categories for easier comparison. The diagnosis groups were meningitis, encephalitis, meningoencephalitis, brain abscess and other CNS infections.

Patients were regarded as immunocompromised if they used medication or suffered from an underlying disease that might affect their immune status, or if the article stated they were immunocompromised.

Pleocytosis was defined as having ≥ 5 cells/µL in CSF or if the article self-reported pleocytosis. Biochemical parameters for CSF included protein level, glucose level and/or lactate. These parameters were regarded as abnormal if above or below defined thresholds (Supplementary Table S3), or if the article self-reported them as abnormal.

## Results

### Articles included

**Fig. 1:**
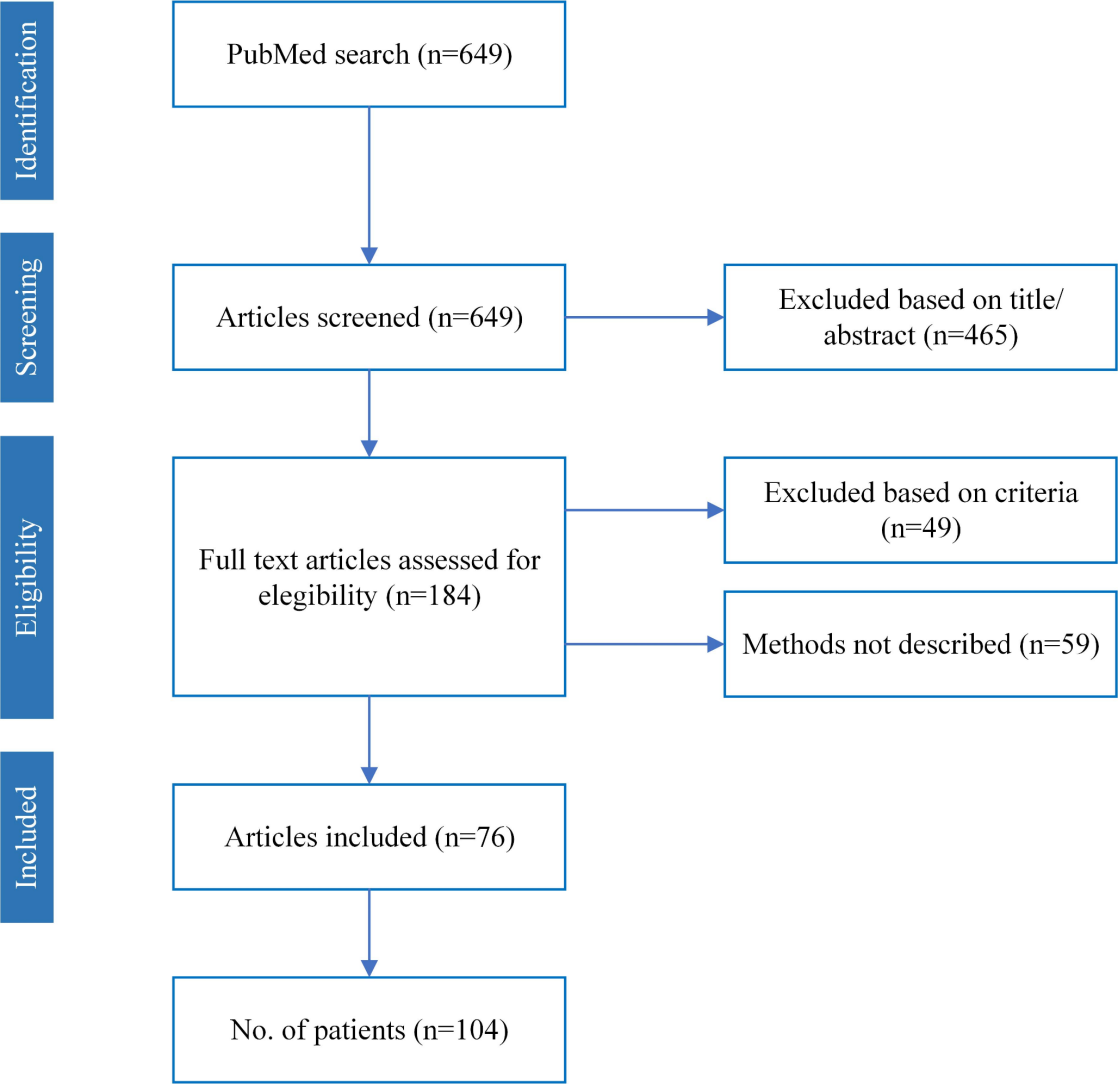
Flowchart of the systematic review of case reports

The PubMed search identified 649 articles, of which, 465 were excluded based on a title/abstract screening (Fig. 1). Of the 184 articles assessed in full text, 49 were eliminated according to the exclusion criteria. Additionally, 59 of the 184 articles were termed “methods not described”. These papers met the inclusion criteria, but their methods were insufficiently reported, or it was unclear whether they used untargeted mNGS. Data from these articles is available in Supplementary Table S4 but was not further analysed. In total, 76 articles were included in the data extraction and analysis (Table 1). The articles originated from 12 different countries; the majority came from China (59%) and the USA (13%) (Table 1). More than half of the articles (67%) were published between 2020 and 2024.

**Table 1:** Included case reports by microorganism and pathogen name

Author		Ref.	Publication year	No. of cases	Sample	Micro-organism	Category	Pathogen Name	Broad diagnosis	Immuno-compromised	Gender	Age	Pleocytosis	Abn. biochem	Sequencing technology	Country
	**Bacteria**															
Tian, Y.		[36]	2022	1	CSF	Bacteria	Gram neg	*Acinetobacter baumannii*	Other CNS infection	No	F	15	Yes	Yes	Illumina	China
Zhang, H.		[37]	2023	1	CSF	Bacteria	Gram neg	*Bordetella pertussis*	Other CNS infection	No	M	0	Yes	Yes	Illumina	China
Yu, L.		[38]	2023	8	CSF	Bacteria	Gram neg	*Brucella spp.*	Other CNS infection	No	M	62	Yes	Yes	Illumina	China
					CSF	Bacteria	Gram neg	*Brucella spp.*	Meningitis	No	M	32	Yes	Yes	Illumina	China
					CSF	Bacteria	Gram neg	*Brucella spp.*	Other CNS infection	No	M	29	Yes	Yes	Illumina	China
					CSF	Bacteria	Gram neg	*Brucella spp.*	Meningoencephalitis	No	M	15	Yes	Yes	Illumina	China
					CSF	Bacteria	Gram neg	*Brucella spp.*	Meningitis	No	M	28	Yes	Yes	Illumina	China
					CSF	Bacteria	Gram neg	*Brucella spp.*	Meningitis	No	M	51	Yes	Yes	Illumina	China
					CSF	Bacteria	Gram neg	*Brucella spp.*	Meningoencephalitis	No	M	44	Yes	Yes	Illumina	China
					CSF	Bacteria	Gram neg	*Brucella spp.*	Other CNS infection	No	M	70	No	No	Illumina	China
Shi, Y.		[20]	2021	1	CSF	Bacteria	Gram neg	*Chlamydia psittaci*	Meningitis	No	F	54	Yes	Yes	Illumina	China
Shi, Q.		[39]	2021	1	CSF	Bacteria	Gram neg	*Escherichia coli*	Other CNS infection	No	F	N/A	Yes	Yes	Illumina	China
Ding, M		[40]	2023	4^†^	CSF	Bacteria	Gram neg	*Haemophilus influenzae*	Meningitis	No	F	9	Yes	Yes	BGI	China
Wilson, M. R.		[41]	2014	1	CSF	Bacteria	Gram neg	*Leptospira*	Other CNS infection	Yes	M	14	Yes	Yes	Illumina	USA
Zhang, X.		[42]	2022	1	CSF	Bacteria	Gram pos	*Listeria monocytogenes*	Encephalitis	Yes	M	39	Yes	Yes	Illumina	China
Lan, Z. W.		[43]	2020	1	CSF	Bacteria	Gram pos	*Listeria monocytogenes*	Meningoencephalitis	No	F	66	Yes	Yes	Illumina	China
Zhang, X.		[44]	2021	1	CSF	Bacteria	Gram pos	*Listeria monocytogenes*	Meningoencephalitis	No	M	64	Yes	Yes	Illumina	China
Yu, L.		[45]	2023	5	CSF	Bacteria	Gram pos	*Listeria monocytogenes*	Meningoencephalitis	Yes	F	23	Yes	Yes	Illumina	China
					CSF	Bacteria	Gram pos	*Listeria monocytogenes*	Meningoencephalitis	Yes	M	56	Yes	Yes	Illumina	China
					CSF	Bacteria	Gram pos	*Listeria monocytogenes*	Meningoencephalitis	No	F	53	Yes	Yes	Illumina	China
					CSF	Bacteria	Gram pos	*Listeria monocytogenes*	Meningoencephalitis	No	M	61	Yes	Yes	Illumina	China
					CSF	Bacteria	Gram pos	*Listeria monocytogenes*	Meningoencephalitis	No	M	50	Yes	Yes	Illumina	China
Courbin, V.		[46]	2022	1	CSF	Bacteria	Gram pos	*Nocardia cyriacigeorgica*	Other CNS infection	Yes	M	66	Yes	Yes	Illumina	France
Zhou, C.		[47]	2021	1	CSF	Bacteria	Gram pos	*Nocardia farcinica*	Brain abcsess	Yes	M	50	Yes	Yes	Illumina	China
Pan, L.		[48]	2021	1	CSF	Bacteria	Gram pos	*Nocardia farcinica*	Other CNS infection	No	M	72	N/A	N/A	BGI	China
Wylie, K.M.		[49]	2016	1	CSF	Bacteria	Gram pos	*Propionibacterium acnes*	Meningitis	Yes	M	40	Yes	Yes	Illumina	USA
Ding, M		[40]	2023	4^†^	CSF	Bacteria	Gram neg	*Pseudomonas aeruginosa*	Meningitis	Yes	M	3	No	No	BGI	China
Zeng, Z.		[50]	2021	1	CSF	Bacteria	Gram neg	*Rickettsia felis*	Encephalitis	No	F	26	Yes	No	BGI	China
Ding, M		[40]	2023	4^†^	CSF	Bacteria	Gram pos	*Staphylococcus aureus*	Meningitis	No	M	9	Yes	Yes	BGI	China
Horiba, K.		[51]	2021	2	CSF	Bacteria	Gram pos	*Streptococcus agalactiae*	Meningitis	No	M	0	Yes	Yes	Illumina	Japan
							Gram pos	*Streptococcus agalactiae*	Meningitis	No	M	0	No	No	Illumina	Japan
Gao, L. Y.		[52]	2020	1	CSF	Bacteria	Gram pos	*Streptococcus intermedius*	Brain abcsess	No	M	55	Yes	Yes	Ion Proton	China
Zhang, J.		[52]	2022	1	CSF	Bacteria	Gram pos	*Streptococcus intermedius*	Meningitis	No	M	11	Yes	Yes	N/A	China
Ding, M.		[40]	2023	4^†^	CSF	Bacteria	Gram pos	*Streptococcus pneumoniae*	Meningitis	No	M	0	Yes	Yes	BGI	China
Liu, C.		[53]	2022	1	CSF	Bacteria	Gram neg	*Treponema pallidum*	Other CNS infection	No	M	29	Yes	Yes	Illumina	China
Zhan, C.		[54]	2021	1	CSF	Bacteria	Mycoplasma	*Ureaplasma parvum*	Meningitis	No	M	0	Yes	Yes	BGI	China
	**Fungi**															
Mao, Y.		[17]	2021	1	CSF	Fungus	Ascomycetes	*Coccidioides posadasii*	Meningitis	No	F	49	Yes	Yes	Illumina	China
Wang, D. M.		[16]	2020	1	CSF	Fungus	Ascomycetes	*Talaromyces marneffei*	Other CNS infection	No	F	33	Yes	Yes	BGI	China
Zhu, Y. M.		[15]	2018	1	CSF	Fungus	Ascomycetes	*Talaromyces marneffei*	Other CNS infection	No	M	22	No	Yes	BGI	China
	**Parasites**															
Feng, L.		[55]	2020	1	CSF	Parasite	Helminth	*Angiostrongylus cantonensis*	Meningitis	No	M	27	Yes	Yes	Illumina	China
Liu, J.		[55]	2022	1	CSF	Parasite	Helminth	*Angiostrongylus cantonensis*	Meningitis	No	M	8	Yes	Yes	Illumina	China
Xie, M.		[56]	2019	2	CSF	Parasite	Helminth	*Angiostrongylus cantonensis*	Meningoencephalitis	No	M	1	Yes	N/A	BGI	China
							Helminth	*Angiostrongylus cantonensis*	Meningoencephalitis	No	M	1	Yes	N/A	BGI	China
Yang, Y.		[57]	2020	1	CSF	Parasite	Amoeba	*Balamuthia mandrillaris*	Encephalitis	No	M	2	Yes	Yes	Illumina	China
Hirakata, S.		[58]	2021	1	BT	Parasite	Amoeba	*Balamuthia mandrillaris*	Encephalitis	Yes	F	60	Yes	Yes	Illumina	Japan
Wilson, M. R.		[59]	2015	1	CSF	Parasite	Amoeba	*Balamuthia mandrillaris*	Meningoencephalitis	Yes	F	74	Yes	Yes	Illumina	USA
Wang, Q.		[60]	2018	1	CSF	Parasite	Amoeba	*Naegleria fowleri*	Meningoencephalitis	No	M	42	Yes	Yes	BGI	China
Huang, S.		[61]	2021	1	CSF	Parasite	Amoeba	*Naegleria fowleri*	Meningoencephalitis	No	M	8	Yes	Yes	Illumina	China
Guan, Q.		[62]	2022	1	CSF	Parasite	Amoeba	*Naegleria fowleri*	Meningoencephalitis	No	M	59	N/A	Yes	Illumina	Saudi Arabia
Liu, P.		[63]	2018	1	CSF	Parasite	Helminth	*Taenia solium*	Other CNS infection	No	F	44	Yes	Yes	BGI	China
Chen, B.		[64]	2021	1	CSF	Parasite	Helminth	*Taenia solium*	Other CNS infection	No	F	27	Yes	Yes	Illumina	China
Pedrosa, D.A.		[65]	2023	1	CSF	Parasite	Helminth	*Taenia solium*	Meningitis	No	F	68	Yes	Yes	Illumina	Brazil
Hu, Z.		[66]	2018	1	CSF	Parasite	Protozoa	*Toxoplasma gondii*	Encephalitis	Yes	M	31	Yes	No	BGI	China
Yu, C.W.		[67]	2023	1	CSF	Parasite	Protozoa	*Toxoplasma gondii*	Encephalitis	No	N/A	3	Yes	Yes	MGI	China
	**Viruses**															
Brown, J. R.		[68]	2015	1	BT	Virus	ssRNA(+)	Astrovirus VA1/HMO-C	Encephalitis	Yes	M	1	N/A	N/A	Illumina	UK
Frémond, M. L.		[69]	2015	1	BT	Virus	ssRNA(+)	Astrovirus VA1/HMO-C	Encephalitis	Yes	M	14	No	No	Ion Proton	France
Naccache, S. N.		[70]	2015	1	BT	Virus	ssRNA(+)	Astrovirus VA1/HMO-C	Encephalitis	Yes	M	42	No	Yes	Illumina	USA
Lum, S. H.		[71]	2016	1	BT	Virus	ssRNA(+)	Astrovirus VA1/HMO-C	Encephalitis	Yes	F	0	No	Yes	Illumina	UK
Hurley, S.		[72]	2023	1	BT	Virus	ssRNA(-)	Avian paramyxovirus type 1	Encephalitis	Yes	F	2	N/A	N/A	Illumina	Australia
Wilson, M.R.		[73]	2017	1	CSF	Virus	ssRNA(-)	Cache Valley virus	Meningoencephalitis	Yes	M	34	Yes	No	Illumina	USA
Kang, Y.		[74]	2021	1	CSF	Virus	ssRNA(+)	Echovirus 18	Encephalitis	No	M	4	Yes	N/A	Illumina	China
Huang, L.		[75]	2021	1	CSF	Virus	dsDNA	Epstein-Barr virus	Encephalitis	No	M	59	Yes	Yes	Illumina	China
Regnault, B.		[76]	2022	1	BT	Virus	ssRNA(-)	European bat lyssavirus 1	Encephalitis	No	M	59	Yes	Yes	Illumina	France
Perlejewski, K.		[77]	2015	1	CSF	Virus	dsDNA	Herpes simplex 1	Encephalitis	No	M	60	Yes	Yes	Illumina	Poland
Zhang, Y.		[78]	2019	1	CSF	Virus	dsDNA	Herpes simplex 1	Encephalitis	Yes	F	47	No	Yes	BGI	China
Liu, L. L.		[79]	2020	1	CSF	Virus	dsDNA	Herpes simplex 1	Encephalitis	No	M	1	Yes	Yes	BGI	China
Osterman, A.		[80]	2020	1	BT	Virus	dsDNA	Herpes simplex 1	Encephalitis	No	F	48	Yes	Yes	Illumina	Germany
Guan, H.		[81]	2016	4^†^	CSF	Virus	dsDNA	Herpes simplex 1	Meningoencephalitis	No	M	64	Yes	Yes	BGI	China
							dsDNA	Herpes simplex 1	Meningoencephalitis	No	M	41	Yes	Yes	BGI	China
							dsDNA	Herpes simplex 2	Meningoencephalitis	No	M	31	Yes	Yes	BGI	China
Olson, C.A.		[82]	2019	1	CSF	Virus	dsDNA	Human herpesvirus 6B	Encephalitis	No	M	1	No	No	Illumina	USA
Li, S.		[83]	2022	1	CSF	Virus	dsDNA	Human Herpesvirus 7	Encephalitis	No	M	28	Yes	Yes	Illumina	China
Cao, J.		[84]	2020	1	CSF	Virus	ssDNA	Human parvovirus B19	Encephalitis	No	M	17	No	Yes	Illumina	China
Yu, S.		[85]	2023	1	CSF	Virus	ssRNA(-)	Human respiratory syncytial virus	Encephalitis	No	M	12	N/A	N/A	Illumina	China
Solomon, I. H.		[86]	2021	1	CSF	Virus	ssRNA(-)	Jamestown Canyon virus	Encephalitis	Yes	M	56	Yes	Yes	Illumina	USA
Maamary, J.		[87]	2023	1	BT	Virus	ssRNA(+)	Japanese encephalitis virus	Meningoencephalitis	No	M	70*	Yes	Yes	Illumina	Australia
Rodriguez, C.		[88]	2020	1	BT	Virus	ssRNA(-)	Measles virus	Encephalitis	Yes	F	28	No	No	Illumina	France
Huang, M.		[89]	2019	1	CSF	Virus	dsDNA	Molluscum contagiosum virus	Other CNS infection	No	F	25	No	Yes	BGI	China
Morfopoulou, S. D.		[90]	2017	1	BT	Virus	ssRNA(-)	Mumps virus	Encephalitis	Yes	M	1	Yes	Yes	Illumina	UK
Solomon, I. H.		[91]	2018	1	CSF	Virus	ssRNA(+)	Powassan virus	Encephalitis	Yes	M	63	Yes	Yes	Illumina	USA
Farrington, M.		[92]	2023	1	CSF	Virus	ssRNA(+)	Powassan virus	Encephalitis	No	M	4	Yes	No	Illumina	USA
Liu, E.		[93]	2022	1	CSF	Virus	ssRNA(-)	Seoul orthohantavirus	Other CNS infection	Yes	M	13	N/A	Yes	Illumina	China
Chiu, C.Y.		[94]	2017	1	CSF	Virus	ssRNA(+)	St. Louis encephalitis virus	Meningoencephalitis	Yes	M	68	Yes	No	Illumina	USA
Zhou, Y.		[95]	2022	2	CSF	Virus	dsDNA	Suid herpesvirus 1	Encephalitis	No	F	51	No	No	BioelectronSeq4000	China
						Virus	dsDNA	Suid herpesvirus 1	Encephalitis	No	M	68	Yes	Yes	BioelectronSeq4000	China
Ikuta, Y.		[96]	2019	1	CSF	Virus	ssDNA	Torque teno virus	Meningitis	No	M	0	Yes	No	Illumina	Japan
Cordey, S.		[97]	2015	1	CSF	Virus	ssRNA(-)	Toscana virus	Meningitis	No	M	61	Yes	Yes	Illumina	Switzerland
Tschumi, F.		[98]	2019	1	CSF	Virus	ssRNA(-)	Toscana virus	Meningitis	No	M	23	Yes	Yes	Illumina	Switzerland
Pérot, P.		[99]	2021	2	BT	Virus	ssRNA(-)	Umbre orthobunyavirus	Encephalitis	Yes	M	21	No	No	Illumina	France
							ssRNA(-)	Umbre orthobunyavirus	Encephalitis	Yes	F	58	Yes	Yes	Illumina	France
El-Duah, P.		[100]	2022	1	CSF	Virus	dsDNA	Varicella-zoster virus, Human immunodeficiency virus	Encephalitis	No	M	11	No	No	Illumina	Ghana
Guan, H.		[81]	2016	4^†^	CSF	Virus	dsDNA	Varicella-zoster virus	Meningoencephalitis	No	F	46	Yes	Yes	BGI	China
Fang, M.		[101]	2020	1	CSF	Virus	dsDNA	Varicella-zoster virus	Other CNS infection	Yes	M	28	Yes	Yes	BGI	China
Chen, L.		[102]	2020	4	CSF	Virus	dsDNA	Varicella-zoster virus	Meningitis	No	M	52	Yes	Yes	Illumina	China
							dsDNA	Varicella-zoster virus	Meningitis	No	M	22	Yes	Yes	Illumina	China
							dsDNA	Varicella-zoster virus	Meningitis	No	M	45	Yes	Yes	Illumina	China
							dsDNA	Varicella-zoster virus	Meningitis	No	M	29	Yes	Yes	Illumina	China
Xie, Z.		[103]	2021	1	CSF	Virus	dsDNA	Varicella-zoster virus	Other CNS infection	Yes	M	41	Yes	Yes	BioelectronSeq4000	China
Han, J.		[104]	2023	5	CSF	Virus	dsDNA	Varicella-zoster virus	Encephalitis	No	M	66	Yes	Yes	BGI	China
							dsDNA	Varicella-zoster virus	Encephalitis	No	M	41	Yes	Yes	BGI	China
							dsDNA	Varicella-zoster virus	Encephalitis	No	F	65	Yes	Yes	BGI	China
							dsDNA	Varicella-zoster virus	Encephalitis	No	M	72	Yes	Yes	BGI	China
							dsDNA	Varicella-zoster virus	Encephalitis	No	M	75	Yes	Yes	BGI	China
Wilson, M. R.		[105]	2017	1	CSF	Virus	ssRNA(+)	West Nile Virus	Meningoencephalitis	Yes	F	14	Yes	Yes	Illumina	USA

CSF- cerebrospinal fluid, BT– Brain tissue, F– female, M– male, N/A– not available, Abn. biochem - abnormal biochemical analysis in CSF. *Reported to be in his 70’s. ^†^Names of microorganisms sorted alphabetically; therefore, the cases appear accordingly

### Patient characteristics

The 76 articles included described 104 patients, of which 77 were male (75%). For one of the patients, the gender was not specified. The median age was 31.5 years (IQR: 11–56 years) for all patients, wereas the median age for male patients was 32 (IQR: 13–56 years), and 46 (IQR: 25–54 years) for female patients. The age was not reported for one of the female patients and one of the male patients was denoted as in his 70’s (Table 1).

**Fig. 2:**
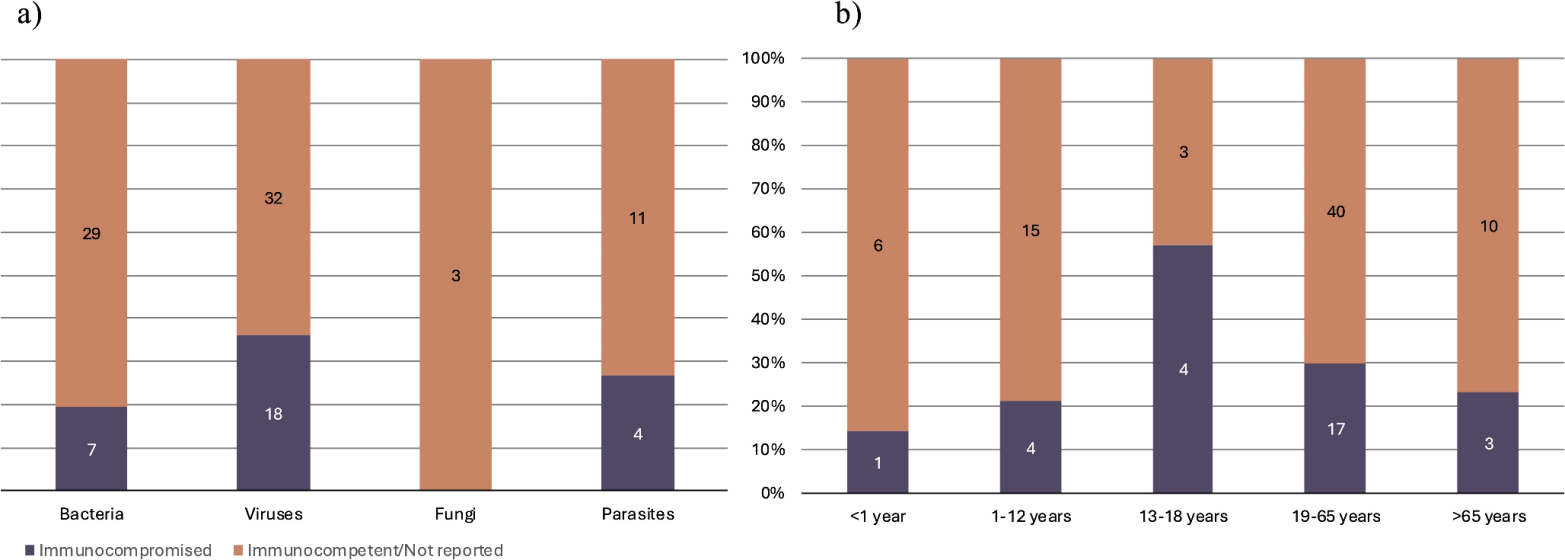
Distribution of immunocompromised patients according to (**a**) infecting pathogen and (**b**) age. Age was not reported for one of the patients

Of all patients, 28% were considered immunocompromised. The proportion was 25% (19/77) among male patients and 35% (9/26) among female patients. The distribution of immune status according to infecting pathogen and age is presented in Fig. 2.

In 98 of the cases, the CSF cell count was reported, and of these 83 (85%) displayed pleocytosis. In 97 patients, the authors reported one or more biochemical parameters in CSF, of these, 82 (85%) had abnormal biochemical results (Table 1).

In 12 cases, there was a reported travel history prior to the infection. All four groups of pathogens were represented in these patients, but viral infections were most common (8/12, 67%).

### Diagnosis

Encephalitis was the most common diagnosis, affecting 37 patients (36%). This was followed by meningitis in 24 patients (23%) and meningoencephalitis in 23 patients (22%). The remaining patients were diagnosed with brain abscesses (*n* = 2, 2%) and other CNS infections (*n* = 18, 17%) (Table 1).

### Samples and methods

Most clinical samples were CSF (*n* = 91, 88%), whereas the remaining cases were brain tissue (*n* = 13, 12%). In more than half of the patients, only DNA was isolated from the samples (*n* = 69, 66%), while others only isolated RNA (*n* = 21, 20%) and 11% both DNA and RNA (*n* = 11). Three articles did not specify the extraction of nucleic acids (3%). Illumina was the most frequent sequencing technology used (54/76 articles), followed by technology from BGI (16/76) (Table 1).

### Pathogens

**Fig. 3:**
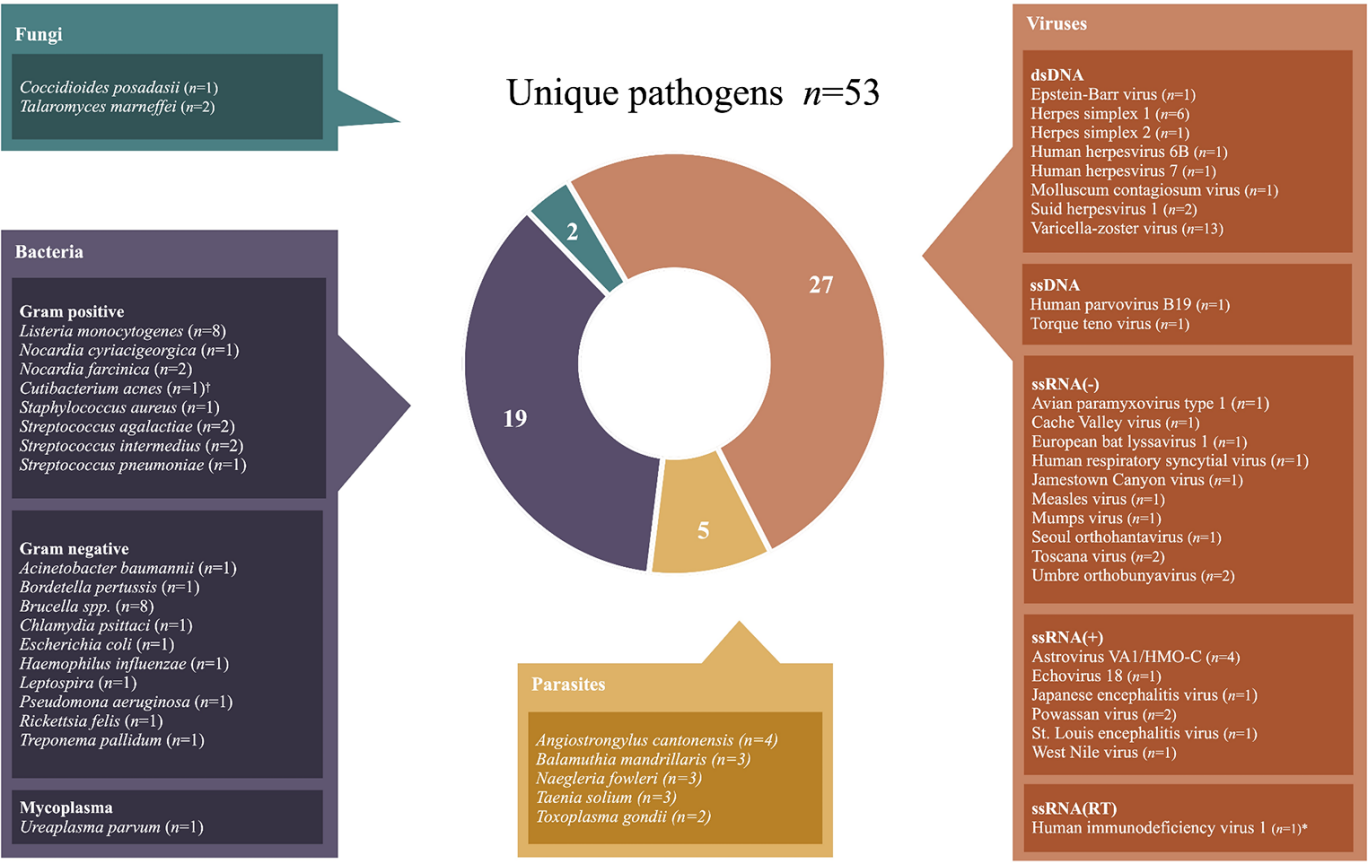
Pathogens identified with mNGS in patients with CNS infections. *One patient had a co-infection with VZV and HIV. ^†^Formerly known as *Propionibacterium acnes*

Among the 104 patients, 105 pathogens were identified, with one patient experiencing a co-infection of two pathogens. These pathogens comprised 53 unique species: 27 viruses, 19 bacteria, 5 parasites, and 2 fungi (Fig. 3; Table 1). The most frequent pathogens detected were varicella-zoster virus (*n* = 13, 12%), *Brucella spp.* (*n* = 8, 8%), *Listeria monocytogenes* (*n* = 8, 8%), herpes simplex virus 1 (*n* = 6, 6%), VA-HMO astrovirus (*n* = 4, 4%) and *Angiostrongylus cantonensis* (*n* = 4, 4%).

**Fig. 4:**
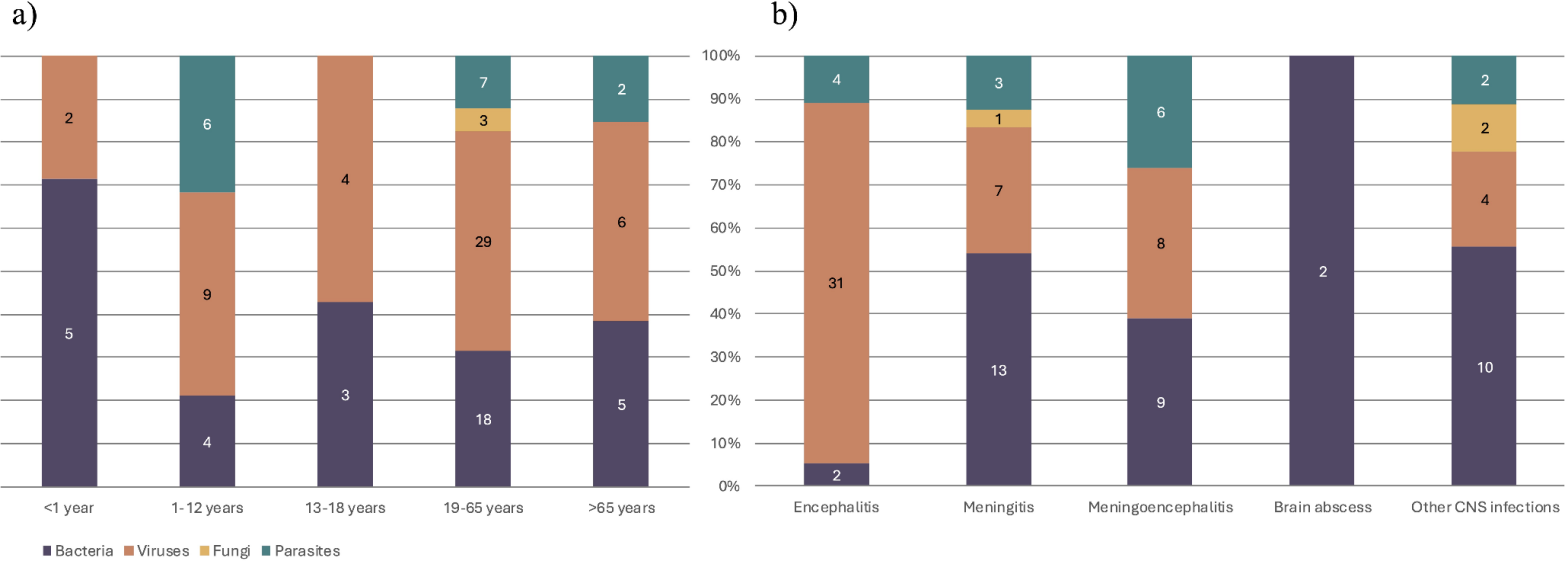
Distribution of infection causing pathogens per patient according to (**a**) age and (**b**) diagnosis groups. Age was not reported for one of the patients

Figure 4 illustrates the distribution of pathogens by age and diagnosis. Most patients (57 out of 103) were between 19 and 65 years old, and half of this group was infected by a virus. Among patients diagnosed with encephalitis, 84% had viral infections (Fig. 4b).

## Discussion

This systematic review highligts the transformative role of mNGS in diagnosing CNS infections. As an advanced diagnostic tool, mNGS surpasses traditional methods by quickly and accurately identifying a wide range of pathogens. Our review illustrates its effectiveness in clinical settings, identifying 53 different pathogens across 104 patients of varying ages and immune statuses.

Immunocompromised patients are more prone to infections, especially opportunistic ones, including fungal infections [11]. The percentage of immunocompromised patients with CNS infection varies across studies, depending on the specific diagnosis and study population [2, 12, 13], and it has been shown that mNGS is particulary useful for detecting patogens in these patients [14]. Interestingly, the three cases of fungal CNS infections identified in this review were not reported as immunocompromised [15–17]. Additionally, the results from this review underline that this method would also be a powerful tool for the immunocompetent host, as more than two-thirds of the patients were not reported as being immunocompromised.

Although rare, encephalitis and meningitis are the most prevalent infections in the CNS. These were the most frequent diagnoses among the case reports, with encephalitis at 36% and meningitis at 23%. The combination of the two, meningoencephalitis, followed at 22%. Not surprisingly, the cases of encephalitis were mainly caused by viruses (31/37 patients) and overall, viral CNS infections accounted for almost half of the cases (50/104 patients). This review identified 27 different viruses, ranging from common ones, such as HSV1/2 and VZV, to more rare or unexpected viruses. Toscana virus, Jamestown Canyon virus, Japanese encephalitis virus, Powassan virus, Cache Valley virus and West Nile virus are examples that show geographic variations. The wide range of viruses adds to the complexity of the aetiology of CNS infections, stressing the need for a more comprehensive assay. Syndromic panels for meningitis/encephalitis [18] would only have detected four of the 27 viruses. Therefore, mNGS is a useful tool for broadening the detection range. Notably, only about 30% of the samples isolated RNA, and a mere 11% included both RNA and DNA. Omitting RNA from the lab protocol would exclude many potential pathogens, since more than half of the viruses in this study are RNA viruses.

For the patients with a bacterial infection, 19 different species were identified in our review. Of these, 14 can be characterised as slow-growing or fastidious. While culture remains the gold standard for detecting bacterial infections, serology is a better option for some hard-to-culture species like *Treponema pallidum*, *Rickettsia felis* and *Leptospira sp.* However, serology testing has several drawbacks: lack of antibody response in immunosuppressed patients, the need for prior pathogen suspicion and potential non-specific reactions. For some bacteria, such as *Listeria monocytogenes*, gram stain and culture sensitivity can be low [19]. For infections like *Chlamydia psittaci* [20] - an obligate intracellular bacterium causing psittacosis - a specific PCR is necessary. However, not all diagnostic laboratories offer this test, due to the rarity of respiratory psittacosis in humans.

Parasites can be difficult to detect by conventional techniques, and although these infections are rare, they can cause serious illness. For instance, *Balamuthia mandrillaris* is associated with high mortality [21]. The identification of the amoeba is difficult and it is rarely detected in CSF samples. Moreover, the diagnosis is often established *postmortem* [21]. Not all parasitic infections are as severe as *B mandrillaris*. The nematode *Angiostrongylus cantonensis* is often the cause of eosinophilic meningitis, and the disease varies from mild to fatal [22, 23]. Also for the helminth *Taenia solium*, where the larval stage causes neurocysticercosis (NCC), the clinical manifestation and the severity of the disease, greatly vary [24]. Diagnosing the aforementioned parasites can be challenging as it relies on clinical symptoms or targeted analysis. However, all 15 cases of parasitic CNS infections reported here exemplify how mNGS offers a potential for rapid and accurate diagnosis.

There were three patients with a fungal CNS infection. Two were infected by *Talaromyces marneffei* and one with *Coccidioides posadasii. T. marneffei* is mainly found in patients with weakened immunity who live in or have traveled to southeast Asia [25]. The diagnosis depends on culture from either CSF or other specimens, if disseminated manifestation of talaromycosis [26]. However, as for other fungi and fastidous organisms, this is slow and the sensitivity is suboptimal [27]. *C. posadasii* also displays a geographic distribution and is endemic to certain parts of the USA, Central and South America [28]. The case included in this review was a Chinese woman who had recently travelled to Los Angeles, USA. This highlights how travel history can broaden the range of suspected causative pathogens.

The application of mNGS for CNS infections has become increasingly popular, as is evident by the rise in published case reports over the past decade. A recent publication demonstrates the method’s effectiveness in clinical practice and highlights its broad capacity to identify a vast array of pathogens [29]. Moreover, its utility extends beyond the mere identification of pathogens; accurate genotyping can aid in outbreak investigations and provide crucial information on virulence factors and resistance genes [30], aiding in the treatment and diagnosis of patients. Despite its diagnostic promise, mNGS still faces challenges in clinical adoption. Key issues include standardising wet and dry lab protocols, interpreting low-read results, and reducing the time from sample collection to result [30, 31]. Significant efforts have been made to standardise both laboratory processes for mNGS in clinical settings [32, 33]. Reducing the turnaround time is crucial in infectious medicine, and advancements in technology for real-time analysis show promising results [34, 35]. Notably, none of the included studies in our review used this technology.

There are limitations to this review. Case reports are often published because the authors have encountered unusual cases or information, and do not necessarily represent a typical patient or situation. Some reports are published due to a rare pathogen or diagnosis, others because they have used a novel method to detect a conventional CNS pathogen. Nevertheless, looking at a larger number of case reports using mNGS, can help form an image of the use of mNGS in a clinical setting and highlight the advantages.

For some articles, it was difficult to assess if it was mNGS or not, as some only stated “second generation sequencing”, “NGS” or “mNGS” with no additional description of the methods. As the term “metagenomics” can entail both targeted and untargeted approaches, we decided to exclude these. The 59 articles excluded due to this criterion were all published in 2019 and later, and the lack of description is possibly because mNGS has become a more common tool for CNS infections and the authors do not see the need for further elaboration.

## Conclusion

In conclusion, this systematic review underscores the pivotal role of mNGS in diagnosing CNS infections, highlighting its potential to revolutionise clinical diagnostics by offering an unparalleled breadth of pathogen detection. As mNGS technology advances and becomes more integrated into clinical practice, its ability to detect rare and fastidious pathogens will be instrumental in improving patient outcomes, particularly in complex and high-stakes cases like CNS infections.

## Electronic supplementary material

Below is the link to the electronic supplementary material.

Supplementary Material 1

## Data Availability

No datasets were generated or analysed during the current study.
